# Barriers and facilitators to implementing laparoscopic surgery in low- and middle-income countries: a systematic review using the consolidated framework for implementation research

**DOI:** 10.1007/s00464-026-13414-z

**Published:** 2026-10-13

**Authors:** Ali Yasen Mohamedahmed, Mohammed A. Adam, Seddig Adam Jebril Saleh, Malaz A. E. Dafallah, Omar Elfaroug Salim, Kayla Bagg, Johnelize Louw, Tiffany E. Chao, Kathryn Chu

**Affiliations:** 1 Centre for Global Surgery, Stellenbosch University, Stellenbosch, South Africa; 2 Department of General and Hepatobiliary Surgery, University Hospitals of Leicester, Leicester, UK; 3 Clinical Service of HPB Surgery and Liver Transplantation, Royal Free Hospital NHS Foundation Trust, London, UK; 4 Department of General Surgery, University of Al Fashir, Al Fashir, Sudan; 5 Department of General Surgery, Elrazi University, Khartoum, Sudan; 6 Department of Surgery, College of Medicine – Ajman University, Ajman, UAE; 7 Department of Surgery, Stanford University School of Medicine, Stanford, CA USA

**Keywords:** Laparoscopic surgery, Implementation science, Low- and middle-income countries, Healthcare innovation, CFIR framework

## Abstract

**Background:**

Although laparoscopic surgery delivers substantial clinical benefits, its adoption across low- and middle-income countries (LMICs) remains inconsistent and incomplete. This systematic review aims to synthesise the barriers and facilitators of laparoscopic surgery adoption within LMICs healthcare systems using the Consolidated Framework for Implementation Research (CFIR).

**Methods:**

A comprehensive systematic search was conducted in PubMed, MEDLINE, ScienceDirect, Embase, and Scopus to identify studies reporting barriers and facilitators to the adoption of laparoscopic surgery in LMICs, as defined by the World Bank classification. Data were extracted using standardised forms, and all implementation factors were mapped to CFIR domains. Additionally, a systematic thematic analysis was then conducted to refine and interpret the extracted data.

**Results:**

Across 18 studies, 417 factors were identified, including 222 barriers and 195 facilitators. Predominant barriers were high equipment costs (*n* = 24, 11%), structural limitations (*n* = 18, 8%), and limited training capacity (*n* = 27, 12%). Key facilitators included perceived clinical advantage (*n* = 26, 13%), institutional leadership engagement (*n* = 18, 9%), international collaboration (*n* = 13, 7%), and technical adaptability (*n* = 11, 6%). Inner-setting factors were most frequently represented (*n* = 114, 27%), reflecting institutional readiness as a critical determinant.

**Conclusions:**

Implementation of minimally invasive surgery in LMICs is shaped by barriers including infrastructure and training limitations and facilitators such as leadership, collaboration, and adaptability—with inner-setting factors most frequent, indicating that strengthening institutional readiness is pivotal. Strategic deployment of international partnerships and locally tailored implementation pathways can accelerate equitable access to this essential surgical innovation.

**Supplementary Information:**

The online version contains supplementary material available at https://doi.org/10.1007/s00464-026-13414-z.

Globally, about 5 billion people lack access to safe, affordable surgical and anaesthesia care, and an estimated 321.5 million additional procedures are needed annually, with shortfalls concentrated in LMICs [1]. Within this context, minimally invasive surgery (MIS) can reduce postoperative morbidity and length of stay, yet adoption in LMICs remains uneven [2]. Understanding the specific barriers and facilitators to implementing laparoscopy (a major MIS modality) is essential to guide equitable scale-up [3].

The clinical benefits of MIS over open surgery are well established: smaller incisions reduce tissue trauma, resulting in 20–30% shorter recovery, less postoperative pain, and shorter hospital stays. MIS is also associated with lower surgical site infection rates and earlier return to normal activities [4], which are the benefits that provide particular value in resource-constrained settings where prolonged admissions and wound complications carry high patient and system costs [5].

Despite these benefits, the adoption of laparoscopy has been highly variable across different economic and geographical contexts. While HICs have routinely integrated laparoscopic techniques into standard surgical practice, low- and middle-income countries (LMICs) have experienced “sporadic and marginal adoption” [6]. Understanding the barriers and facilitators to laparoscopy in LMICs is needed to improve its implementation [7].

Implementation of laparoscopic surgery in LMICs faces complex barriers beyond resources, including high costs, personnel shortages, infrastructure issues, cultural resistance, and limited support. Wilkinson et al. 2021. identified seven main challenges: funding, equipment, trainers, stakeholder dynamics, curricula, departmental structure, and opportunities for trainees [3].

The Consolidated Framework for Implementation Research (CFIR), developed by Damschroder et al. [8], is widely used to understand the factors that affect the adoption, implementation, and sustainability of evidence-based practices across healthcare settings. It organises factors into five domains: innovation characteristics, outer setting, inner setting, characteristics of individuals, and process.

We conducted a systematic review using the Consolidated Framework for Implementation Research (CFIR) to identify and synthesise barriers and facilitators to implementing laparoscopy in LMICs. We focus on laparoscopy as a core modality within MIS and use “laparoscopy” thereafter, except when referring to MIS generically. MIS literature in LMICs is growing; prior reviews largely emphasise technical aspects, training, or clinical outcomes and have not systematically examined multilevel implementation barriers and facilitators within a formal framework [3]. By structuring evidence within CFIR, this review provides actionable insights for healthcare leaders, policymakers, and researchers seeking to expand equitable access to laparoscopic surgery in resource-limited settings [9].

## Methods

This systematic review was conducted in accordance with the Preferred Reporting Items for Systematic Reviews and Meta-Analyses (PRISMA) 2020 guidelines [10, 11]. This study formed part of a broader prospectively registered systematic review examining barriers and facilitators, patient outcomes, and costs associated with gastrointestinal endoscopy and minimally invasive general surgery in LMICs (PROSPERO CRD42024593992). The present analysis focuses specifically on the prespecified objective concerning barriers and facilitators to the implementation of laparoscopic surgery.

### Search strategy and inclusion criteria

The search strategy encompassed both database searches and supplementary methods to ensure comprehensive identification of the literature. A systematic search was conducted across multiple electronic databases, including PubMed, MEDLINE, ScienceDirect, Embase, and Scopus. Moreover, search engines such as Google Scholar were used to identify additional eligible studies. The literature search was performed using a combination of Medical Subject Headings (MeSH) terms and free-text keywords related to barriers, facilitators, and the implementation of laparoscopic surgery in LMICs. The search combined three main concepts: (laparoscopic/minimally invasive surgery) AND (implementation barriers/facilitators) AND (LMIC/resource-limited settings). The full database-specific search strategies are presented in Supplementary Table 1. Additionally, the reference list of relevant articles was screened to identify potentially eligible articles published from 1996 to 2024 and written in English, as resources for reliable translation of non-English full texts were unavailable.

### PICOS eligibility criteria

**Population (P)**: Healthcare professionals, surgical teams, healthcare institutions, or patients involved in the provision or implementation of laparoscopic surgery in low- and middle-income countries (LMICs).

**Intervention/exposure (I)**: Implementation, introduction, adoption, delivery, or use of laparoscopic/minimally invasive surgery within LMIC healthcare settings.

**Comparator (C)**: No comparator was required. Where applicable, studies comparing laparoscopic surgery with open surgery, different settings, or different implementation periods were eligible if they provided relevant implementation data.

**Outcomes (O)**: Reported barriers, facilitators, challenges, enablers, determinants, or other factors influencing the implementation, adoption, uptake, delivery, or sustainability of laparoscopic surgery.

**Study design (S)**: Quantitative, qualitative, and mixed-methods studies providing data relevant to the implementation of laparoscopic surgery in LMICs.

### Selection criteria

Studies were eligible for inclusion if they reported on implementation barriers or facilitators of laparoscopic surgery within clinical settings. Only studies published in the English language and that were conducted in countries classified by the World Bank as LICs, LMICs, or UMICs during the year of publication were considered for inclusion. To ensure methodological rigour, inclusion was limited to those that presented primary empirical data, such as observational, cross-sectional, cohort, or mixed-methods research, conducted in real-world healthcare contexts. Studies were excluded if they were non-English publications, from high-income countries, conference abstracts, or did not present a complete methodological description. Research not specifically addressing laparoscopic surgery, for example, studies reported on general minimally invasive techniques or other surgical innovations, was also excluded. Additionally, studies were not eligible if they failed to report barriers or facilitators related to the implementation of laparoscopic surgery.

### Methodological quality assessment

The methodological quality of the included studies was assessed using the Mixed-Methods Appraisal Tool (MMAT), 2018 version. [12] The MMAT enables critical appraisal of empirical studies across five methodological categories: qualitative research, randomised controlled trials, non-randomised studies, quantitative descriptive studies, and mixed-methods studies. Following two initial screening questions, studies were classified by design and assessed against the five corresponding MMAT criteria. Each criterion was rated as “Yes”, “No”, or “Can’t tell” based on the information reported in the study.

### Data extraction and use of CFIR

Data extraction was conducted using a standardised and piloted extraction form developed specifically for this review. Three reviewers independently extracted data from each included study to ensure completeness and accuracy, with discrepancies resolved by consensus or a fourth reviewer (AS). The extraction process was managed using DistillerSR (Systematic Review & Literature Review Software by DistillerSR Inc), which enabled systematic organisation and verification of data. Extracted information included study identification details (author, year, and country or region), study design and methodology, population and sample characteristics, details of the surgical intervention or implementation context, and comprehensive descriptions of barriers and facilitators.

All extracted barriers and facilitators were categorised according to the five domains of the CFIR [8]: intervention characteristics, inner setting, outer setting, characteristics of individuals, and process. This framework-based approach ensured a consistent and systematic categorisation of barriers and facilitators, enabling meaningful comparisons and synthesis of implementation factors across diverse study designs and settings. To enhance clarity and facilitate reproducibility, the CFIR domains were explicitly operationalised for the context of laparoscopic surgery implementation in LMICs (Table S2).

### Data synthesis and analysis

Given the heterogeneity of study designs, populations, and implementation contexts, a narrative qualitative synthesis was undertaken to integrate findings across the included studies. The synthesis process followed established guidance for qualitative evidence synthesis, incorporating principles of thematic analysis to identify recurrent patterns, themes, and relationships among reported implementation factors. This approach enabled a comprehensive understanding of the diverse influences shaping the adoption of laparoscopic surgery in LMICs.

A systematic thematic analysis [13] was then conducted to refine and interpret the extracted data. The analysis followed the six established phases: familiarisation with the data; generation of initial codes; identification and development of themes; review and refinement of emerging themes; definition and naming of final themes; and synthesis and reporting of findings using the CFIR framework. This process helped identify key themes and patterns, showing how barriers and facilitators work together and how factors at the individual, organisational, and wider healthcare system levels affect the implementation of laparoscopic surgery.

### Statistical analysis

Descriptive analyses were used to summarise the characteristics of the included studies and the identified implementation factors. Categorical data were summarised using frequencies and percentages, including the overall counts of barriers and facilitators, as well as their distribution across CFIR domains and constructs. The frequency of reporting was used to describe how commonly individual implementation factors were identified in the included evidence and was not interpreted as a measure of their relative importance or the magnitude of their effects. Given the heterogeneity of study designs, populations, and implementation contexts, and the qualitative nature of the implementation factors, no meta-analyses were performed.

## Results

**Fig. 1:**
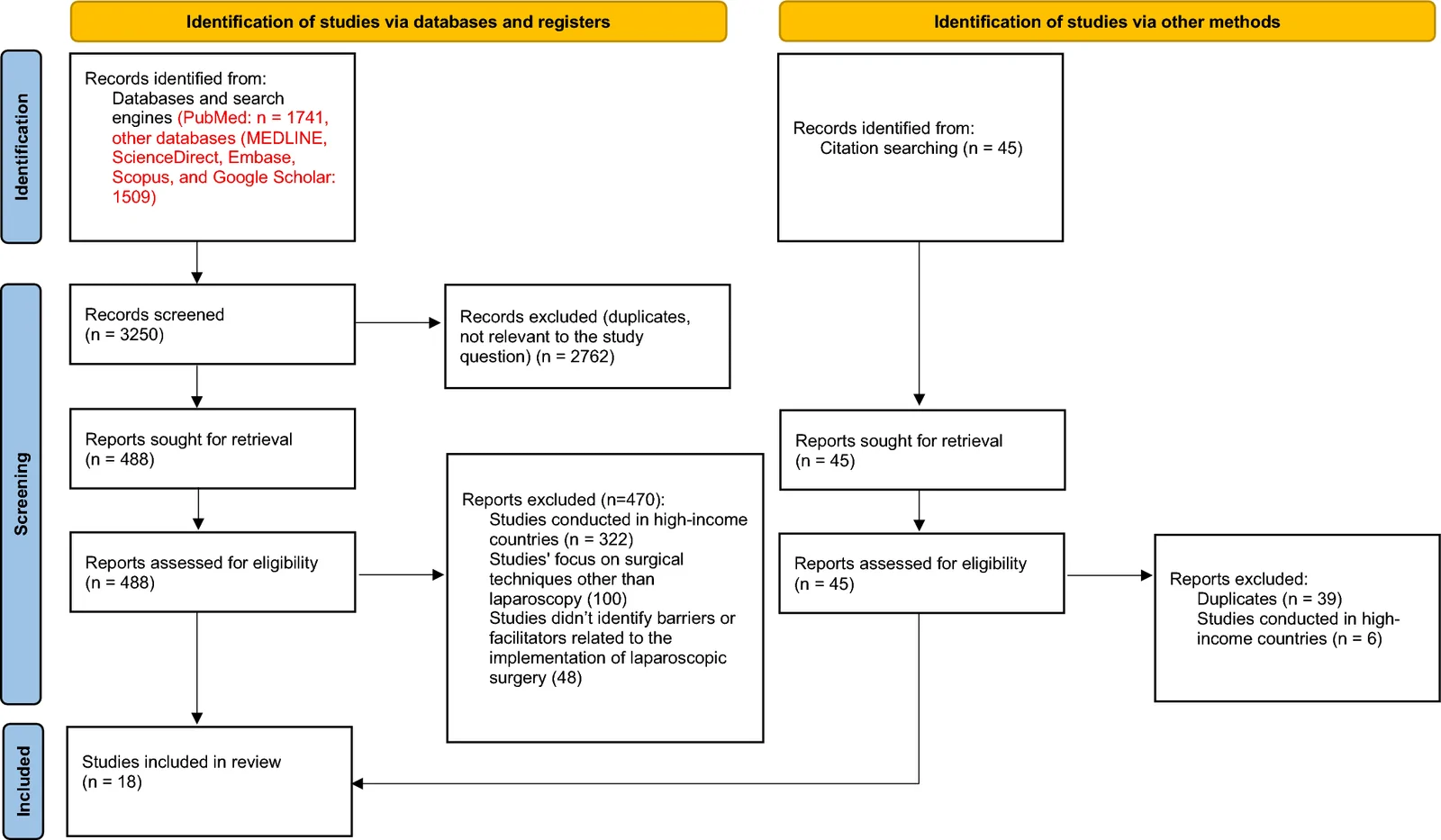
PRISMA flow diagram

A total of 18 studies [14–31] were included in this review following the screening of 3,250 database records and 45 additional sources (Fig. 1). The studies spanned a wide geographic distribution across LMICs, including the African region, the Region of the Americas, the South-East Asia Region, the Eastern Mediterranean Region, and the Western Pacific Region. Characteristics of the included studies are shown in Table 1.

**Table 1:** Baseline characteristics of the included studies

Study	Country		Participants	Details of participants and study methodology
Nyundo et al. [14]	East, Central, Southern Africa	Cross-sectional study	Surgeons in COSECSA-accredited training hospitals	94 surgeons from 44 hospitals in 16 countries
Choy et al. [15]	Ghana	Exploratory case study	Surgeons at a large teaching or referral hospitals	600 h of participant observation as well as 13 in-depth interviews (4 senior surgeons, 4 residents, 3 nurses, 1 anaesthesiologist,1pharmacist) and document analyses, were collected over 12 weeks
Imran et al. [16]	Guatemala	Observational study	General surgeons at Hospital Nacional de San benito	7 general surgeons were interviewed, and 9402 cholecystectomies over 14 years were reviewed
Tanoli et al. [17]	LMIC	Survey-based study	Healthcare professionals (surgeons, trainees, biomedical engineers) in LMIC	34 participants from 25 countries (79.4% surgical consultants, 14.7% surgical trainees, 2.9% biomedical engineers
Damous et al. [18]	Brazil	Retrospective cohort study	Analysis of the clinical data of patients undergoing emergency appendectomy at a tertiary public hospital	1168 laparoscopic appendectomies between 2004 and 2018
Rombaldi et al. [19]	Brazil	Cross-sectional survey study	Paediatric surgeons in Brazil	187 surgeons (General paediatric surgery: 70.6%, Paediatric urology: 18.2%, Surgical oncology: 7%, Thoracic surgery: 4%)
Gupta et al. [20]	India	Retrospective cohort study	Analysis of clinical data of patients undergoing elective laparoscopic cholecystectomy	145 patients (86% females, 13% males) who underwent laparoscopic cholecystectomy
Misauno [21]	Nigeria	Prospective cohort study	Analysis of clinical data of patients undergoing laparoscopic cholecystectomy	35 patients (86% females, 14% males)
Robertson et al. [22]	Rwanda	Mixed-methods study	Logbook of surgeons and survey for surgical staff in four national referral hospitals	Twenty-two interviews for surgeons and 209 laparoscopic procedures were reviewed (27.3% general surgery and 72.7% gynaecological procedures)
Afuwape and Akute [31]	Nigeria	Perspective article	NA	General observations from University College Hospital, Ibadan, Nigeria
Yankunze et al. [23]	East, Central, Southern Africa	Retrospective cross-sectional study	Analysis of the surgical logbook of COSECSA general surgery trainees	68,659 cases reviewed and 616 were laparoscopic from 194 trainees
Reynolds et al. [24]	Liberia	Mixed-methods study (survey and interview)	Liberian surgeons and surgical trainees	Survey: 31 participants Interviews: 15 participants (interns = 2, residents/fellows = 5, consultants = 8)
Asbun et al. [25]	Bolivia	case study	Local general surgeons, operating room staff, anaesthesiologists, nurses	8 surgeons fully trained/proctored basic laparoscopy, 7 surgeons trained in advanced procedures, 35 medical/paramedical staff attended didactics
Bryce-Alberti et al. [26]	Mexico	Nationwide retrospective analysis	Patients undergoing three common surgical procedures in the Mexican public hospitals: cholecystectomy, appendectomy, inguinal hernia repair	97,234 surgical procedures across 676 public hospitals in all 32 Mexican states
Djote et al. [27]	Ethiopia	Prospective comparative study	Clinical outcomes of patients undergoing elective cholecystectomy for gallstone disease	Total of 119 patients (54.6% Open cholecystectomy, 45.4% Laparoscopic cholecystectomy)
Esmer et al. [28]	Turkey	Cross-sectional survey	Surgeons actively practising in university, public, or private hospitals	Total of 244 surgeons from 55 cities (response rate: 9.2% of 2647 contacted)
Wells et al. [29]	Mongolia	Cross-sectional review	Clinical outcome of patients undergoing laparoscopic cholecystectomy in Mongolia (2005–2013)	Total of 14,522 cholecystectomies from 6 of 21 provinces, including 7 regional diagnostic centres and 2 tertiary academic medical centres
Ndong et al. [30]	Senegal	Review article	Clinical outcome of patients undergoing laparoscopic surgery in Senegal	Analysis of clinical data of 4953 patients who underwent laparoscopic surgery

Study populations included surgeons, surgical trainees, anaesthesiologists, nurses, and biomedical engineers, as well as clinical datasets of patients undergoing laparoscopic procedures. Sample sizes varied considerably, from small exploratory interviews with fewer than 10 participants [15, 25] to large national datasets with more than 90,000 surgical cases [26]. Thematic foci also differed across studies: some examined implementation challenges and the experiences of minimally invasive surgery [14, 22, 24], while others explored the feasibility of laparoscopic procedures [18, 20, 21, 27, 29].

### Methodological quality assessment

Sixteen [14–29] of the 18 included publications were primary empirical studies eligible for MMAT appraisal. Methodological quality was variable. Common concerns among quantitative descriptive studies were representativeness and nonresponse bias, particularly in survey-based studies, while non-randomised studies were limited mainly by residual confounding and retrospective data completeness. The qualitative study demonstrated good methodological coherence and triangulation. Among mixed-methods studies, Reynolds et al. demonstrated the strongest integration and component quality [24]; Robertson et al. [22] study was limited principally by survey nonresponse, and Tanoli et al. [17] study was limited by sampling and representativeness. Two publications [30, 31] were not eligible for MMAT because they fell outside the methodological categories covered by the MMAT. Methodological quality assessment is shown in Supplementary Table 3.

### Barriers and facilitators to the implementation of laparoscopic surgery

**Fig. 2:**
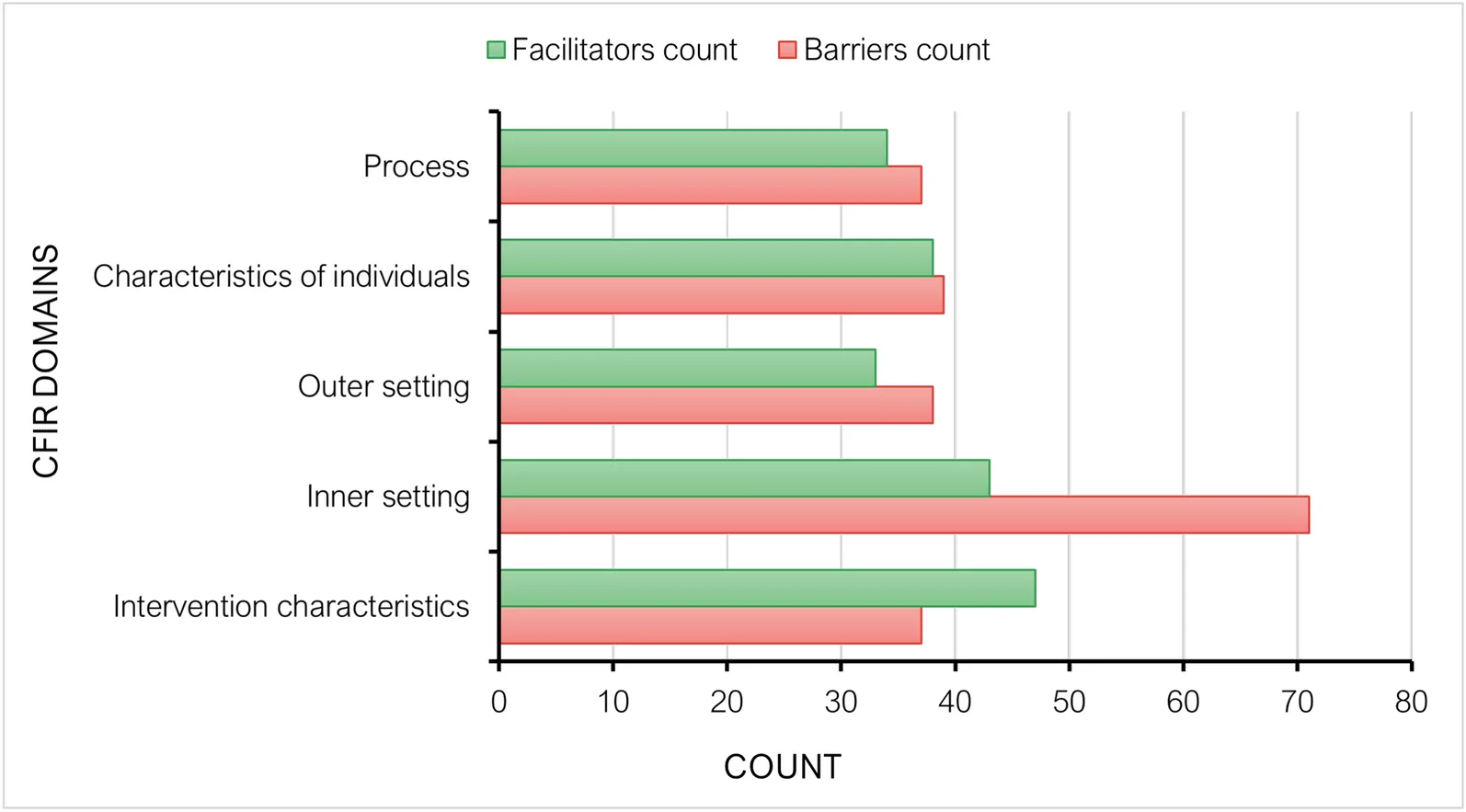
Mapping of reported facilitators and barriers across CFIR domains

**Fig. 3:**
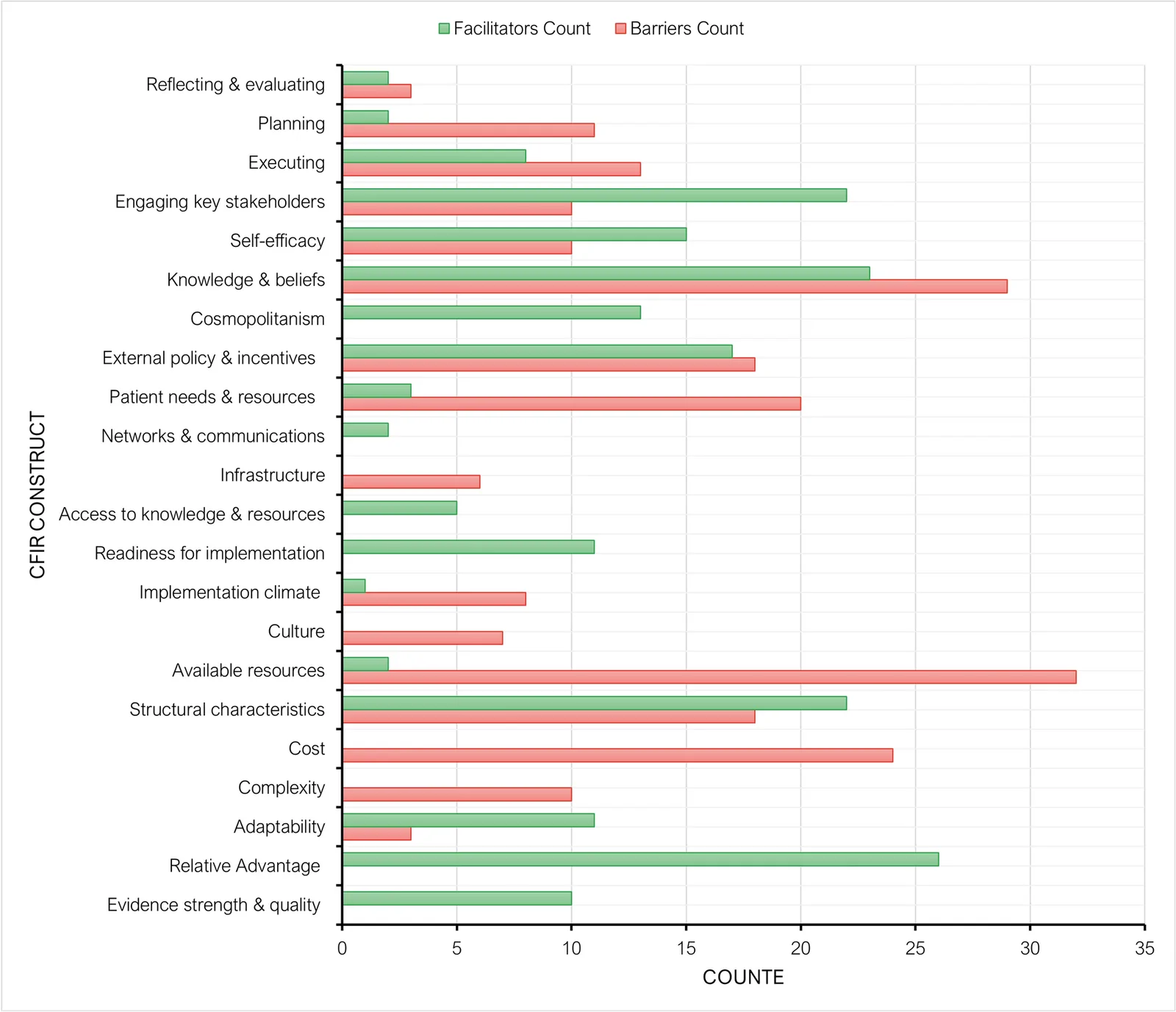
Distribution of reported facilitators and barriers across CFIR constructs

A total of 417 implementation factors were extracted from the 18 included studies, consisting of 222 barriers and 195 facilitators. These factors were mapped across the five CFIR domains (Figs. 2 and 3). The most frequently represented domains were inner setting (*n* = 114), intervention characteristics (*n* = 84), and characteristics of individuals (*n* = 77).

#### Domain 1: intervention characteristics

As illustrated in Fig. 3, this domain captured 84 factors describing attributes of MIS such as evidence strength and quality, relative advantage, adaptability, complexity, and cost. Facilitators included the perceived relative advantage (*n* = 26) of laparoscopy over open surgery, with multiple studies emphasising benefits such as reduced postoperative pain, shorter hospital stays, and faster recovery. Adaptability was reported 14 times overall (11 facilitators and 3 barriers), reflecting how surgical teams modified laparoscopic techniques and equipment to suit limited-resource environments. Cost (*n* = 24) and technical complexity (*n* = 10) were the most frequently cited barriers (Fig. 2). High procurement costs for laparoscopic towers, instruments, and maintenance, combined with limited local suppliers and technical support, restrict availability; inadequate repair facilities and reliance on imported consumables further compound sustainability challenges.

#### Domain 2: inner setting

The inner-setting domain contained the highest number of barriers and facilitators (*n* = 114), underscoring the importance of institutional readiness and infrastructure (Fig. 2).

Internal facilitators were frequently reported (*n* = 43), including supportive organisational culture, strong leadership engagement, and a commitment to innovation. Hospitals with proactive administrative backing and designated MIS champions were more likely to initiate and sustain programmes; multidisciplinary collaboration and peer mentorship embedded in routines further enhanced implementation, as shown in Fig. 3.

Internal barriers such as structural limitations were reported more often (*n* = 71), mainly outdated facilities, inadequate operating theatre space, and unreliable electricity and oxygen supplies. Another key barrier was insufficient resources (*n* = 34), and poor equipment availability also constrained service delivery (Fig. 3).

#### Domain 3: outer setting

The outer setting domain encompassing external policies and incentives, patient needs and resources, and cosmopolitanism (i.e. inter-institutional collaboration) accounted for several facilitators and barriers (*n* = 71). External policies and incentives (*n* = 35) played a dual role, facilitating implementation when governmental or professional bodies endorsed MIS, yet posing barriers when national surgical plans lacked funding or policy frameworks to support technology adoption. Patient needs and expectations (*n* = 23) also influenced uptake; in some regions, limited public awareness and misconceptions about laparoscopic safety reduced demand, increasing patient preference for minimally invasive options, acting as a facilitator, driving hospital-level change.

Cosmopolitanism (*n* = 13) emerged as a facilitator, reflecting international collaborations, visiting surgical teams, and exchange programmes that enhanced skill transfer and technical support.

#### Domain 4: characteristics of individuals

This domain included barriers and facilitators (*n* = 77) that focused on individual-level factors, including knowledge, beliefs, and self-efficacy, among surgeons and surgical teams (Fig. 3). Knowledge and beliefs about laparoscopy were reported 52 times overall (23 facilitators and 29 barriers); positive beliefs reflected recognition of its advantages in patient outcomes and motivation to adopt the technique where feasible, whereas negative beliefs and knowledge gaps acted as barriers. Similarly, self-efficacy was reported 25 times overall (15 facilitators and 10 barriers), with greater confidence in performing laparoscopic procedures supporting adoption. Exposure to hands-on training, mentorship, and prior experience increased individual readiness for implementation. Knowledge gaps, limited exposure during residency, and apprehension about complications were frequently cited barriers. The lack of formal training opportunities in many LMICs led to a reliance on visiting trainers or short-term workshops, which are often insufficient for skill retention.

#### Domain 5: process

The process domain listed barriers and facilitators (*n* = 71) that captured implementation activities including planning, engaging, executing, reflecting and evaluating (Fig. 3). Engaging key stakeholders was reported 32 times overall (22 facilitators and 10 barriers), while executing implementation activities was reported 21 times (8 facilitators and 13 barriers), underscoring the importance of collaborative implementation involving hospital leadership, anaesthesia teams, and biomedical engineers. Successful implementation was often achieved through stepwise integration, starting with basic procedures such as diagnostic laparoscopy or appendectomy before expanding to advanced operations. In contrast, planning was reported 13 times overall (11 barriers and 2 facilitators), while reflecting and evaluating were reported 5 times (3 barriers and 2 facilitators). Poor planning and inadequate evaluation mechanisms hindered progress, particularly in centres where implementation relied on external donations or visiting missions without long-term sustainability planning. Incorporating systematic evaluation and local ownership was associated with sustained success in several case studies.

### Sankey diagram

**Fig. 4:**
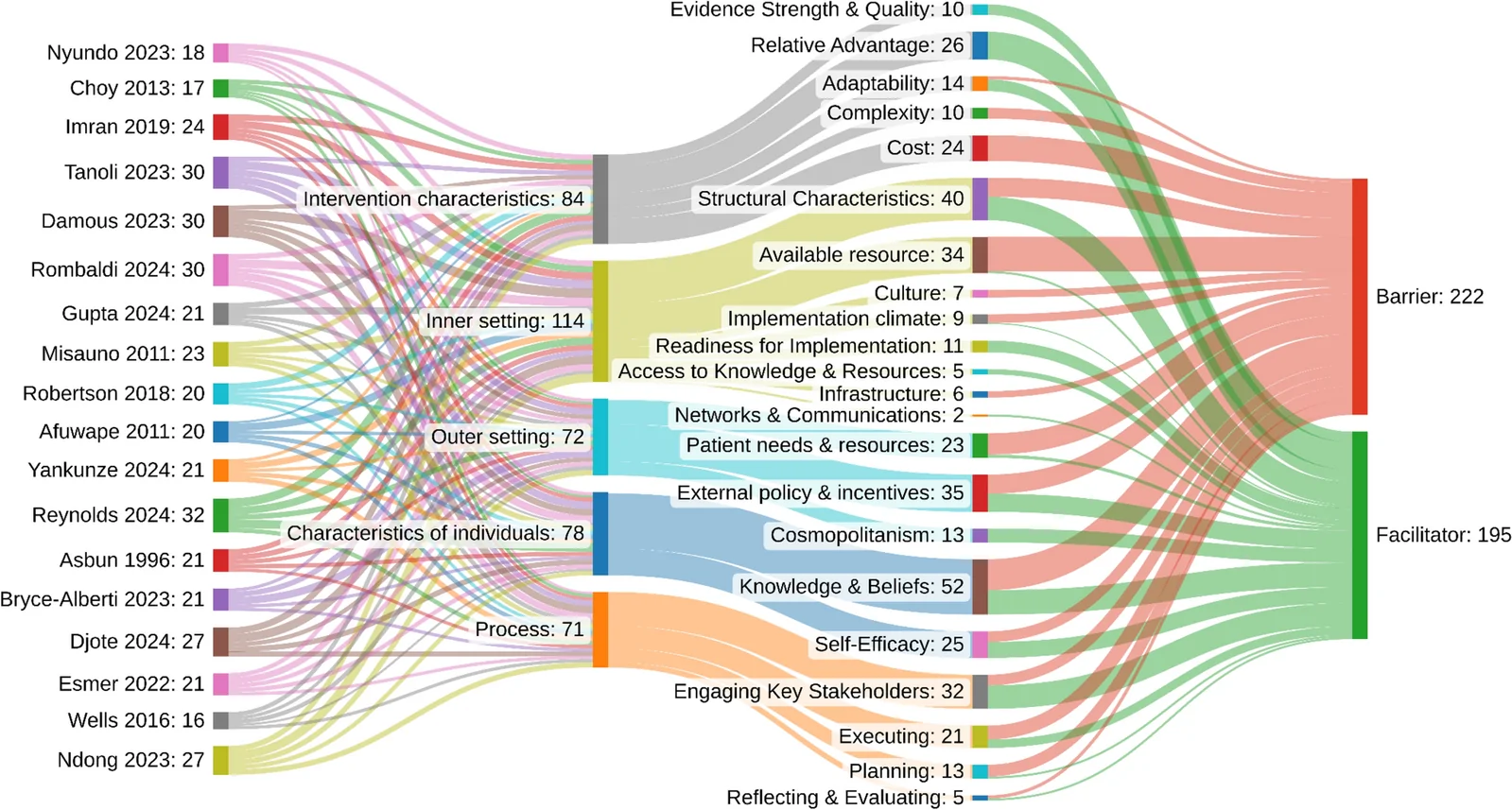
Sankey diagram illustrating barriers and facilitators to the implementation of laparoscopic surgery in low- and middle-income countries (LMICs), mapped using the Consolidated Framework for Implementation Research (CFIR). Each line represents the flow of implementation factors identified across included studies, passing through CFIR domains—intervention characteristics, inner setting, outer setting, characteristics of individuals, and process—to their classification as either barriers (red) or facilitators (green). The width of each band reflects the frequency of reported factors. A total of 417 factors were identified (222 barriers, 195 facilitators) (Color figure online)

Figure 4 presents a Sankey diagram illustrating the distribution and interrelationships of factors influencing the implementation of laparoscopic surgery in LMICs, organised according to CFIR. Each included study (left) contributes data flowing through the five CFIR domains to its classification as a barrier or facilitator on the right. The width of each connecting band represents the frequency with which each factor was reported, allowing for a comparison of their relative influence.

Overall, the figure illustrates that implementation determinants are multifactorial and interdependent, with barriers and facilitators often coexisting within the same domains. While cost, infrastructure, and training limitations remain the primary obstacles, leadership, collaboration, and adaptability consistently emerge as drivers of successful implementation of laparoscopic surgery in LMICs.

## Discussion

This systematic review is the first comprehensive synthesis using formal implementation science methods to identify barriers and facilitators to adoption of minimally invasive surgery in LMICs [8]. Using CFIR, the review goes beyond listing obstacles to reveal multilevel influences on implementation success. Identifying 417 factors across the CFIR domains highlights the complexity of technology diffusion in resource-limited healthcare settings and provides evidence-based guidance for stakeholders seeking to improve surgical capacity [32].

Across all CFIR domains, barriers and facilitators often coexisted within the same construct, reflecting the multifactorial and context-dependent nature of implementing laparoscopic surgery in LMICs. The most prominent barriers related to cost, infrastructure limitations, and inadequate training and resources, whereas key facilitators included institutional support, international collaboration, adaptability of techniques, and positive clinician attitudes. Figures 2 and 3 illustrate the relative distribution of these factors by construct and domain, showing that, while barriers slightly outweighed facilitators, there is growing institutional and professional momentum to expand laparoscopic capacity.

Inner-setting factors highlight that institutional capacity and readiness are key to the adoption of laparoscopy. While technology and evidence are necessary, they are not enough without organisational infrastructure, leadership, and resources. This aligns with broader research showing that successful innovation depends more on organisational readiness than on the innovation itself, as demonstrated across various healthcare contexts [33].

Our synthesis suggests that even evidence-based interventions struggle without organisational readiness. Healthcare leaders should assess institutional capacity and invest in infrastructure, such as electrical systems, oxygen delivery, theatre design, and maintenance, as essential for technology adoption [6, 34]. Without foundational infrastructure, investment in laparoscopic equipment and training becomes underutilised and unlikely to yield lasting clinical benefits.

The pronounced role of cost barriers (*n* = 24) and equipment availability challenges reflects the persistent tension between laparoscopy’s proven clinical benefits and its capital intensity [35]. Conventional procurement models are prohibitively expensive for LMIC institutions with limited budgets, where surgical investments compete with other health priorities such as infectious disease control, maternal health, and primary care expansion [36]. Alternative procurement models should be systematically explored, such as refurbished equipment to extend device lifespan, leasing to spread capital costs, public–private partnerships to leverage resources, regional purchasing to achieve economies of scale, and developing local manufacturing or maintenance capacity and expertise [37, 38]. Furthermore, deliberate engineering innovation for resource-limited contexts, creating lower-cost, locally maintainable laparoscopic systems for environments with unreliable power, limited support, and scarce consumables, is a promising but underexplored area needing more research and development resources [39, 40]. Such innovation might include simplified equipment designs, modular systems for staged procurement, solar- or battery-powered options instead of mains electricity, and simplified sterilisation protocols for limited infrastructure.

Knowledge and beliefs were frequently represented (*n* = 52 overall; 23 facilitators and 29 barriers), indicating that developing human capital is an essential complement to investing in technology and infrastructure rather than a secondary factor [3, 41]. Curricula integration of laparoscopic training, dedicated skills centres, mentorship, and incremental case-based learning effectively enhances surgical education, aligning with adult learning and skill acquisition science [42, 43].

Implementation practitioners should prioritise training pathways tailored to local educational settings, clinician availability, and contextual constraints. Standardised training developed in resource-rich contexts, with protected academic time, abundant simulation, and accessible mentorship, may need significant modification for LMIC healthcare systems, where clinical demands often prevent protected learning time [44, 45]. Moreover, investing in locally embedded training, train-the-trainer models, simulation centres with affordable technology, and integrating laparoscopic training into routine practice through structured apprenticeships offers a sustainable, superior approach to developing human capital than episodic missions or short-term external training. The role of “cosmopolitanism”, or international collaboration mechanisms (*n* = 13), as a facilitator suggests various potential areas for expansion and strategic re-evaluation.

Structured twinning, visiting surgeon exchanges, and virtual mentorships are expandable mechanisms for knowledge transfer, adaptable to various technologies and connectivity environments [46, 47]. South–south collaboration, in which LMIC centres with laparoscopic programmes mentor similar-resource centres, may be more culturally suited than traditional north–south partnerships, which often impose high-income-country practices on resource-constrained environments [48]. Regional networks with multiple LMIC centres can share equipment, coordinate training, benchmark progress, and advocate for policy support and funding, achieving more than isolated efforts.

This review advances surgical implementation science by applying the CFIR to identify determinants of the diffusion of complex surgical technologies [48]. Applying CFIR to laparoscopic surgery shows that it effectively organises complex factors across different system levels, but also highlights unique contextual aspects of LMIC surgical systems that the original framework, developed in high-income countries, doesn’t fully address [49, 50]. CFIR’s standard form provides limited focus on LMIC health system financing, the roles of external advocacy groups and agencies in implementation, and diaspora clinicians linking high- and low-income countries’ contexts [51]. Future LMIC-specific implementation science frameworks could better address unique contextual factors in resource-limited healthcare, improving their usefulness for practitioners’ systems [52]. Applying implementation science frameworks to surgical innovation in LMICS is an emerging research area with the potential to improve evidence-based surgical care.

### Limitations

Several study-specific limitations need acknowledgement to contextualise the evidence and guide future research. First, the 18-study sample size, though substantial for implementation science reviews and reflecting the field’s early stage, may underrepresent some LMIC regions. It may reflect Latin American and Asian experiences more than those in sub-Saharan Africa, where the challenges of laparoscopic surgery may differ substantially [53]. Second, systematic reviews of qualitative and mixed-methods research involve interpretive processes. Three independent reviewers used standardised data extraction forms and resolved discrepancies through discussion to improve reliability. However, qualitative data categorisation and CFIR domain mapping remain subject to reviewer interpretation and potential misclassification despite these measures of rigour [54].

Third, publication bias may skew results towards success stories and established programmes; failures and barriers, though informative, are less often published, possibly inflating the perceived balance of facilitators and barriers [55].

Fourth, the review’s English-language restriction excludes relevant literature in other languages, especially extensive French and Portuguese literature from Latin American contexts, Spanish publications, and African languages, thereby limiting the representativeness of the geographic and contextual scope [56]. Fifth, changes in technology costs, healthcare financing, and infrastructure investment from 1996 to 2024 may limit comparability across studies; early studies reflect technology and cost barriers that have been reduced by innovation, while recent studies may not yet show long-term sustainability patterns for follow-up [57, 58]. Laparoscopy may be particularly valuable in LMICs, where prolonged hospitalisation and wound complications impose high patient and system costs; however, its comparative impact relative to HICs is context-dependent and not established by this review [7]. Finally, the frequency of reported barriers and facilitators should not be interpreted as their relative importance. Frequency counts reflect patterns of reporting rather than the magnitude or impact of implementation determinants.

## Conclusion

This CFIR-guided systematic review identified infrastructure deficits, limited training capacity, and high equipment costs as predominant barriers, whereas perceived clinical advantage, leadership engagement, technical adaptability, and international collaboration were key facilitators. These findings underscore that strengthening institutional readiness, reliable utilities and theatres, equipment availability/maintenance, and structured training pipelines while leveraging leadership and partnerships is central to sustainable scale-up of laparoscopy. Programmes that combine affordable procurement models with stepwise roll out, stakeholder engagement, and routine evaluation are most likely to reduce barriers, amplify facilitators, and translate evidence into equitable uptake.

## Supplementary Information

Below is the link to the electronic supplementary material.

Supplementary file1 (DOCX 18 KB)

Supplementary file2 (DOCX 20 KB)

Supplementary file3 (DOCX 22 KB)

Supplementary file4 (DOCX 270 KB)

## Data Availability

All data relevant to this study are included in the article. Additional data are available on request from the corresponding author.
