# Supplementary material for: Barriers and facilitators to implementing laparoscopic surgery in low- and middle-income countries: a systematic review using the consolidated framework for implementation research
Source: Surg Endosc. 2026 Oct 13;40(11):9418–30. doi: 10.1007/s00464-026-13414-z (PMC13755120; doi:10.1007/s00464-026-13414-z)
Supplement: Supplementary file 1 [file 464_2026_13414_MOESM1_ESM.docx]

**Supplementary Table 1:** Full database-specific search strategies.

| Database | Controlled vocabulary | Free-text search |
| --- | --- | --- |
| PubMed | “Laparoscopy”[Mesh]  “Minimally Invasive Surgical Procedures”[Mesh]  “Developing Countries”[Mesh]  “Diffusion of Innovation”[Mesh]  “Health Plan Implementation”[Mesh] | (  "Laparoscopy"[Mesh]  OR "Minimally Invasive Surgical Procedures"[Mesh]  OR laparoscop*[Title/Abstract]  OR "laparoscopic surgery"[Title/Abstract]  OR "minimally invasive surgery"[Title/Abstract]  OR "minimally invasive surgical procedure*"[Title/Abstract]  )  AND  (  "Diffusion of Innovation"[Mesh]  OR "Health Plan Implementation"[Mesh]  OR implement*[Title/Abstract]  OR adopt*[Title/Abstract]  OR uptake[Title/Abstract]  OR diffusion[Title/Abstract]  OR barrier*[Title/Abstract]  OR challenge*[Title/Abstract]  OR obstacle*[Title/Abstract]  OR facilitator*[Title/Abstract]  OR enabler*[Title/Abstract]  OR determinant*[Title/Abstract]  OR "capacity building"[Title/Abstract]  OR sustainab*[Title/Abstract]  OR feasibility[Title/Abstract]  )  AND  (  "Developing Countries"[Mesh]  OR "developing countr*"[Title/Abstract]  OR "low income countr*"[Title/Abstract]  OR "middle income countr*"[Title/Abstract]  OR "low-income countr*"[Title/Abstract]  OR "middle-income countr*"[Title/Abstract]  OR "low- and middle-income countr*"[Title/Abstract]  OR LMIC[Title/Abstract]  OR LMICs[Title/Abstract]  OR "resource-limited"[Title/Abstract]  OR "resource limited"[Title/Abstract]  OR "resource-constrained"[Title/Abstract]  OR "low-resource setting*"[Title/Abstract]  ) |
| MEDLINE | Laparoscopy/  Minimally Invasive Surgical Procedures/  Developing Countries/  Diffusion of Innovation/  Health Plan Implementation/ | 1. Laparoscopy/  2. Minimally Invasive Surgical Procedures/  3. (laparoscop* or "laparoscopic surgery" or "minimally invasive surgery").ti,ab.  4. 1 or 2 or 3  5. Diffusion of Innovation/  6. Health Plan Implementation/  7. (implement* or adopt* or uptake or diffusion or barrier* or challenge*  or obstacle* or facilitator* or enabler* or determinant*  or "capacity building" or sustainab* or feasibility).ti,ab.  8. 5 or 6 or 7  9. Developing Countries/  10. ("developing countr*" or "low income countr*" or "middle income countr*"  or "low-income countr*" or "middle-income countr*"  or "low- and middle-income countr*" or LMIC or LMICs  or "resource-limited" or "resource limited"  or "resource-constrained" or "low-resource setting*").ti,ab.  11. 9 or 10  12. 4 and 8 and 11 |
| Embase | 'laparoscopy'/exp  'minimally invasive surgery'/exp  'developing country'/exp  'implementation'/exp | (  'laparoscopy'/exp  OR 'minimally invasive surgery'/exp  OR laparoscop*:ti,ab  OR 'laparoscopic surgery':ti,ab  OR 'minimally invasive surgery':ti,ab  )  AND  (  'implementation'/exp  OR implement*:ti,ab  OR adopt*:ti,ab  OR uptake:ti,ab  OR diffusion:ti,ab  OR barrier*:ti,ab  OR challenge*:ti,ab  OR obstacle*:ti,ab  OR facilitator*:ti,ab  OR enabler*:ti,ab  OR determinant*:ti,ab  OR 'capacity building':ti,ab  OR sustainab*:ti,ab  OR feasibility:ti,ab  )  AND  (  'developing country'/exp  OR 'developing countr*':ti,ab  OR 'low income countr*':ti,ab  OR 'middle income countr*':ti,ab  OR 'low- and middle-income countr*':ti,ab  OR LMIC:ti,ab  OR LMICs:ti,ab  OR 'resource limited':ti,ab  OR 'resource-limited':ti,ab  OR 'resource constrained':ti,ab  OR 'low-resource setting*':ti,ab  ) |
| Scopus |  | TITLE-ABS-KEY(  laparoscop*  OR "laparoscopic surgery"  OR "minimally invasive surgery"  )  AND  TITLE-ABS-KEY(  implement*  OR adopt*  OR uptake  OR diffusion  OR barrier*  OR challenge*  OR obstacle*  OR facilitator*  OR enabler*  OR determinant*  OR "capacity building"  OR sustainab*  OR feasibility  )  AND  TITLE-ABS-KEY(  "developing countr*"  OR "low income countr*"  OR "middle income countr*"  OR "low-income countr*"  OR "middle-income countr*"  OR "low- and middle-income countr*"  OR LMIC  OR LMICs  OR "resource-limited"  OR "resource limited"  OR "resource-constrained"  OR "low-resource setting*"  ) |
| ScienceDirect |  | ("laparoscopic surgery" OR laparoscopy OR "minimally invasive surgery")  AND  (implementation OR adoption OR barriers OR challenges OR facilitators  OR enablers OR "capacity building" OR sustainability OR feasibility)  AND  ("developing countries" OR "low-income countries"  OR "middle-income countries" OR "low- and middle-income countries"  OR LMIC OR LMICs OR "resource-limited" OR "low-resource") |
| Google Scholar |  | "laparoscopic surgery" barriers LMIC  "laparoscopic surgery" facilitators LMIC  "laparoscopy" implementation "developing countries"  "laparoscopy" challenges "low- and middle-income countries"  "minimally invasive surgery" implementation "resource-limited settings"  "laparoscopic surgery" adoption "low-resource settings" |
| Citation searching | Citation searching was undertaken by manually screening the reference lists of included studies and relevant review articles to identify additional potentially eligible studies. | |
