# Supplementary material for: Barriers and facilitators to implementing laparoscopic surgery in low- and middle-income countries: a systematic review using the consolidated framework for implementation research
Source: Surg Endosc. 2026 Oct 13;40(11):9418–30. doi: 10.1007/s00464-026-13414-z (PMC13755120; doi:10.1007/s00464-026-13414-z)
Supplement: Supplementary file 2 [file 464_2026_13414_MOESM2_ESM.docx]

Supplementary Table 2: Context-Specific Operationalisation of CFIR Domains for Laparoscopic Surgery Implementation in LMICs

| **CFIR Domain** | **Generic Definition** | **Operationalised Definition for Laparoscopic Surgery Implementation** |
| --- | --- | --- |
| Intervention Characteristics | Attributes of the innovation being implemented | Properties of laparoscopic surgery as an innovation, including perceived relative advantages (reduced pain, faster recovery, reduced infection rates), adaptability of techniques to resource-limited settings, complexity of equipment and technical requirements, cost of equipment procurement and maintenance, and evidence strength supporting minimally invasive approaches |
| Inner Setting | The organisation or setting where implementation occurs | The hospital, surgical department, or operating theatre unit implementing laparoscopic programmes, encompassing physical infrastructure (theatre layout, equipment availability), institutional readiness (staffing, training capacity), organisational culture and leadership support, clinical governance structures, resource availability (electricity, oxygen, consumables), and multidisciplinary team coordination (surgeons, anaesthetists, nurses, biomedical engineers) |
| Outer Setting | The broader system or policy environment | The national and regional health system context, including governmental policies and funding for surgical innovation, professional body endorsements of minimally invasive surgery, external financing and donor support, patient needs and demand for laparoscopic procedures, international partnerships and exchange programmes, and inter-institutional collaboration networks |
| Characteristics of Individuals | Individual-level factors affecting implementation | Surgeon and surgical team-level factors, including knowledge and beliefs about laparoscopic safety and efficacy, self-efficacy and confidence in performing minimally invasive procedures, prior training and exposure to laparoscopic techniques, motivation for adoption, and professional experience with open vs. minimally invasive approaches |
| Process | Implementation activities and strategies | The deliberate actions taken to implement laparoscopic programmes, including stakeholder engagement and participatory planning, stepwise rollout strategies (starting with diagnostic laparoscopy or simple procedures), training and mentorship pathways, quality assurance and outcome evaluation mechanisms, and sustaining strategies beyond initial implementation phases |
