# Supplementary material for: Barriers and facilitators to implementing laparoscopic surgery in low- and middle-income countries: a systematic review using the consolidated framework for implementation research
Source: Surg Endosc. 2026 Oct 13;40(11):9418–30. doi: 10.1007/s00464-026-13414-z (PMC13755120; doi:10.1007/s00464-026-13414-z)
Supplement: Supplementary file 3 [file 464_2026_13414_MOESM3_ESM.docx]

**Supplementary Table 3**: Methodological quality appraisal using the Mixed Methods Appraisal Tool (MMAT) version 2018. Y; Yes, NA; not applicable, N; No, CT; Can’t tell.

A. Classification of studies

| Studies | S1: Clear research question(s)? | S2: Data address research question(s)? | MMAT category |
| --- | --- | --- | --- |
| Nyundo 2023 (14) | Yes | Yes | Quantitative descriptive |
| Choy 2013 (15) | Yes | Yes | Qualitative |
| Imran 2019 (16) | Yes | Yes | Quantitative descriptive |
| Tanoli 2023 (17) | Yes | Yes | Mixed methods |
| Damous 2023 (18) | Yes | Yes | Quantitative non-randomised |
| Rombaldi 2024 (19) | Yes | Yes | Quantitative descriptive |
| Gupta 2024 (20) | Yes | Yes | Quantitative non-randomised |
| Misauno 2011 (21) | Yes | Yes | Quantitative descriptive |
| Robertson 2018 (22) | Yes | Yes | Mixed methods |
| Yankunze 2024 (23) | Yes | Yes | Quantitative descriptive |
| Reynolds 2024 (24) | Yes | Yes | Mixed methods |
| Asbun 1996 (25) | Yes | Yes | Quantitative descriptive |
| Bryce-Alberti 2023 (26) | Yes | Yes | Quantitative descriptive |
| Djote 2024 (27) | Yes | Yes | Quantitative non-randomised |
| Esmer 2022(28) | Yes | Yes | Quantitative descriptive |
| Wells 2016 (29) | Yes | Yes | Quantitative non-randomised |
| Afuwape 2011 (31) | Yes | NA | Not eligible – perspective/non-empirical |
| Ndong 2023 (30) | Yes | NA | Not eligible – systematic/review article (secondary research) |

B. Qualitative studies

| Study | 1.1 Appropriate qualitative approach? | 1.2 Adequate qualitative data collection? | 1.3 Findings adequately derived from data? | 1.4 Interpretation substantiated by data? | 1.5 Coherence among sources, collection, analysis & interpretation? |
| --- | --- | --- | --- | --- | --- |
| Choy 2013 (15) | Yes | Yes | Yes | Yes | Yes |

C. Quantitative descriptive studies

| Studies | 4.1 Sampling strategy relevant? | 4.2 Sample representative? | 4.3 Measurements appropriate? | 4.4 Risk of nonresponse bias low? | 4.5 Statistical analysis appropriate? |
| --- | --- | --- | --- | --- | --- |
| Nyundo 2023 (14) | Y | CT | CT | CT | Y |
| Imran 2019 (16) | Y | Y | Y | Y | Y |
| Rombaldi 2024 (19) | Y | CT | Y | CT | Y |
| Misauno 2011 (21) | Y | CT | Y | Y | Y |
| Yankunze 2024 (23) | Y | Y | Y | Y | Y |
| Asbun 1996 (25) | CT | CT | Y | CT | Y |
| Bryce-Alberti 2023 (26) | Y | Y | Y | Y | Y |
| Esmer 2022 (28) | Y | N | Y | N | Y |

D. Quantitative non-randomised studies

| Studies | 3.1 Participants representative of target population? | 3.2 Measurements appropriate for exposure/intervention and outcome? | 3.3 Complete outcome data? | 3.4 Confounders accounted for? | 3.5 Intervention/exposure occurred as intended? |
| --- | --- | --- | --- | --- | --- |
| Damous 2023 (18) | Y | Y | Y | CT | Y |
| Gupta 2024 (20) | Y | Y | Y | CT | Y |
| Djote 2024 (27) | Y | Y | Y | CT | Y |
| Wells 2016 (29) | CT | Y | CT | CT | Y |

E. Mixed-methods studies

| Studies | 5.1 Rationale for mixed methods? | 5.2 Components effectively integrated? | 5.3 Integration outputs adequately interpreted? | 5.4 Divergences/inconsistencies addressed? | 5.5 Components meet quality criteria? |
| --- | --- | --- | --- | --- | --- |
| Tanoli 2023 (17) | CT | Y | Y | CT | N |
| Robertson 2018 (22) | Y | Y | Y | Y | N |
| Reynolds 2024 (24) | Y | Y | Y | Y | Y |
